# Residual Kidney Function in Hemodialysis: Its Importance and Contribution to Improved Patient Outcomes

**DOI:** 10.3390/toxins16070298

**Published:** 2024-06-28

**Authors:** Yoshitsugu Obi, Jochen G. Raimann, Kamyar Kalantar-Zadeh, Mariana Murea

**Affiliations:** 1 Division of Nephrology, Department of Medicine, The University of Mississippi Medical Center, Jackson, MS 39216, USA; 2 Renal Research Institute, New York, NY 10065, USA; jochen.raimann@rriny.com; 3 Katz School of Science and Health, Yeshiva University, New York, NY 10033, USA; 4 Tibor Rubin Veterans Affairs Long Beach Healthcare System, Long Beach, CA 90822, USA; kkalantar@lundquist.org; 5 The Lundquist Institute at Harbor, UCLA Medical Center, Torrance, CA 90502, USA; 6 Division of Nephrology, Hypertension, and Kidney Transplantation, University of California Irvine, Orange, CA 92868, USA; 7 Department of Internal Medicine, Section on Nephrology, Wake Forest University School of Medicine, Winston-Salem, NC 27101, USA

**Keywords:** incremental hemodialysis, randomized controlled trial, residual kidney function

## Abstract

Individuals afflicted with advanced kidney dysfunction who require dialysis for medical management exhibit different degrees of native kidney function, called residual kidney function (RKF), ranging from nil to appreciable levels. The primary focus of this manuscript is to delve into the concept of RKF, a pivotal yet under-represented topic in nephrology. To begin, we unpack the definition and intrinsic nature of RKF. We then juxtapose the efficiency of RKF against that of hemodialysis in preserving homeostatic equilibrium and facilitating physiological functions. Given the complex interplay of RKF and overall patient health, we shed light on the extent of its influence on patient outcomes, particularly in those living with advanced kidney dysfunction and on dialysis. This manuscript subsequently presents methodologies and measures to assess RKF, concluding with the potential benefits of targeted interventions aimed at preserving RKF.

## 1. Residual Kidney Function: What It Is

**Figure 1:**
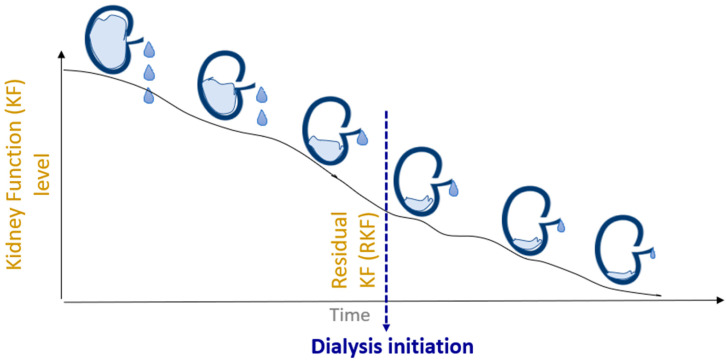
Kidney function before and after dialysis initiation. Residual kidney function (RKF) denotes the level of kidney function present at the time and after dialysis initiation.

Native kidney function involves the concerted functions of multiple components, notably the glomeruli, tubular epithelial cells, and interstitial cells. When the kidney function deteriorates significantly, a time point arises where dialysis becomes a necessary adjunct to existing medical management to uphold the patient’s well-being [1,2]. Most patients have at this point not yet experienced a complete cessation of their native kidney function, and endogenous kidney functionality, known as residual kidney function (RKF), is often present among dialysis patients over extended periods of time (Figure 1).

The extent and duration of this remaining RKF capacity varies significantly between patients, depending largely on the underlying cause and severity of the kidney damage, as well as their overall health and therapeutic management [3]. In this review, we focus on RKF in patients treated with chronic hemodialysis (HD) to underscore the importance of understanding the associated benefits of RKF and the existing options for RKF quantification.

## 2. Residual Kidney Function: Why It Is Important

The dialysis machine, with its critical element being the dialyzer—often referred to colloquially as an artificial kidney—has been engineered with inspiration drawn from the physiological processes in native kidneys [4]. The remarkable scientific achievement embodied in the creation of this synthetic device notwithstanding, it is fundamental to recognize the inherent differences between the dynamic processes occurring within biological systems and those engineered through artificial methodologies and medications. Despite the advancements in dialysis, this treatment assists the organism with certain homeostatic mechanisms rather than fully replacing kidney function [5]. Table 1 compares the key functions of the native kidneys to the corresponding functions executed by HD and accompanying pharmacological interventions, and lists the advantages conferred by residual kidney function.

### 2.1. Uremic Toxin and Acidic Metabolite Clearance

Uremic toxins have been traditionally classified according to their molecular weight as either small (<500 Da) vs. middle (>500 Da) or protein-bound vs. water-soluble (non-protein-bound) [6,7]. Solute clearance by native kidneys involves three mechanisms: glomerular filtration, tubular reabsorption, and tubular secretion. The interplay of these three mechanisms allows the kidneys to perform consistent and constant clearance, selectively reabsorbing essential solutes such as sodium, potassium, calcium, and other small-molecular-weight solutes, while allowing for the excretion of “unnecessary” metabolites (waste products) and excess solutes. In the distal nephron, specialized cells in the collecting ducts either secrete or reabsorb hydrogen and bicarbonate ions, thereby helping to maintain an appropriate systemic pH [8].

Extracorporeal clearance with chronic HD has been designed for the removal of small-molecular-weight solutes and excess water from the blood and is less effective at removing larger solutes, a limitation purposefully technologically enforced to avoid the extraction of larger molecules including albumin and immunoglobulins necessary for vital processes [9]. HD corrects metabolic acidosis by removing surplus acidic metabolites by clearance and adding bicarbonate from the dialysate to neutralize the plasma pH. In fact, in the short term, particularly useful in urgent clinical scenarios, HD is more effective than compromised kidneys in removing electrolytes and other small-molecular-weight molecules during a given period. This can be attributed to the larger surface area of dialyzers, typically ranging between 1.5 and 2.5 m^2^, along with the ability to regulate dialysis parameters like blood and dialysate flow [4]. These factors expedite solute diffusion and removal relative to the significantly reduced total capillary surface area in advanced kidney dysfunction, which is only a fraction of the 0.6 m^2^ found in normal kidneys [10].

**Figure 2:**
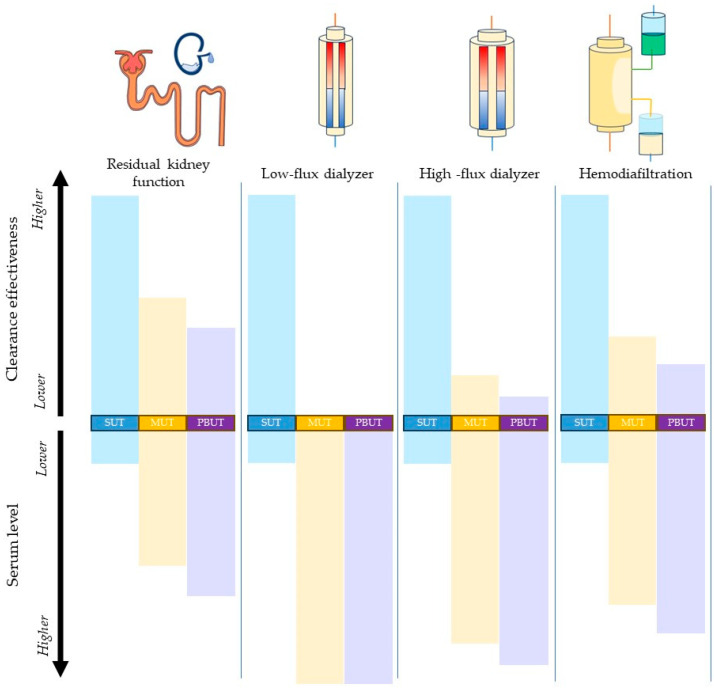
A schematic diagram on the effectiveness of uremic toxin clearance by residual kidney function vs. extracorporeal dialyzer-based modes, along with serum uremic concentrations. The scenarios compared are residual kidney function with a kidney urea clearance (CLurea) of 7 mL/min corresponding to kidney std Kt/V 2.1 (assuming the urea volume of distribution of 35 L) and no extracorporeal clearance vs. extracorporeal urea clearance corresponding to dialysis std Kt/V 2.1 and no residual kidney function. Compared with residual kidney function, all means of extracorporeal clearance are more effective at the removal of non-protein-bound small-molecular-weight uremic toxins (SUTs, denoted in blue rectangles 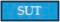). Compared with low-flux dialyzer clearance, high-flux dialyzers and hemodiafiltration are more effective in the removal of medium-molecular-weight uremic toxins (MUTs, denoted in yellow rectangles 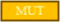) and protein-bound uremic toxins (PBUTs, denoted in purple rectangles 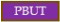), but all are inferior when clearing these molecules compared with residual kidney function.

By comparison, the native kidneys demonstrate a remarkable capability to eliminate large-molecular-weight solutes and protein-bound molecules through endocytosis by tubular epithelial cells [6,11]. Further, the kidneys exhibit an inherent ability to break protein-based bonds, allowing for the liberation, filtration, and thus excretion of protein-bound solutes (Figure 2). Consequently, the native kidneys are uniquely efficient in clearing protein-bound solutes, a significant factor in maintaining homeostatic fluid and electrolyte balance in the body [12].

#### 2.1.1. Small Water-Soluble (Non-Protein-Bound) Uremic Toxins

Urea, phenylacetylglutamine (PAG), trimethylamine-N-oxide protein (TMAO), and guanidines, specifically asymmetric dimethylarginine (ADMA) and symmetric dimethylarginine (SDMA), have been recognized as small water-soluble uremic toxins [13]. Urea, the primary form of nitrogenous waste in the body, was the first marker used to assess kidney function. The acute direct neurotoxicity of urea at clinically relevant levels has been questioned [14]. However, azotemia may contribute to endothelial dysfunction and amino acid depletion by inducing protein carbamylation, leading to the development of atherosclerosis and protein–energy wasting [15]. PAG and TMAO are gut-derived, non-protein-bound uremic toxins suggested as markers and mediators of cardiovascular diseases [7,16,17]. ADMA and SDMA have been shown to have neuro- and cardiovascular toxicity at concentrations typical in uremia. They contribute to the production of pro-inflammatory cytokines such as tumor necrosis factor (TNF)-alpha and interleukin (IL)-6 and inhibit nitric oxide (NO) synthase, leading to endothelial dysfunction and vascular damage. SDMA has been shown to accumulate in HDL particles, thereby enhancing leukocyte activation, promoting reactive oxygen species production, and reducing endothelial NO availability, which could play a role in cardiovascular disease pathogenesis. Plasma levels are highly dependent on glomerular filtration for these small water-soluble uremic toxins except for PAG, the clearance of which depends more on tubular secretion than on glomerular filtration [18].

The contribution of RKF to small water-soluble uremic toxins was demonstrated in a study by Toth-Manikowski et al., who used stored urine and pre-dialysis plasma samples obtained from 1280 HD patients who participated in the Hemodialysis (HEMO) Study. The HEMO Study was a large, national, multicenter, randomized controlled trial investigating dialysis dose and membrane flux [11], where patients with a residual kidney urea clearance (CLurea) ≥ 1.5 mL/min/35 L total body water (TBW) were excluded, since such levels of RKF were considered clinically negligible at that time [19]. Overall, 433 (34%) patients had RKF with an average CLurea of 0.7 mL/min/35 L, and 847 (66%) had no RKF. The levels of PAG, TMAO, ADMA, and SDMA were 15%, 7.4%, 3.7%, and 7.0% lower among patients with RKF vs. no RKF, respectively. Each 0.5 mL/min per 35 L TBW reduction in CLurea was associated with PAG, TMAO, ADMA, and SDMA levels that were 0.67 mg/dL, 6.07 µM, 0.03 µM, and 0.18 µM lower, respectively.

Inorganic phosphate elimination is also significantly enhanced in patients with RKF. A retrospective, cross-sectional study conducted on 79 patients treated with chronic HD observed a strong linear relationship between residual glomerular filtration rate (GFR) calculated via averaging the kidney urea and creatinine clearance from timed urine collection and urinary phosphate excretion [20]. Remarkably, patients possessing a residual GFR of 3 mL/min or above demonstrated approximately double the mean weekly phosphate removal (2000.3 ± 804.1 mg) compared to the amount of phosphate eliminated in a single HD session (1019.9 ± 300 mg, *p* < 0.001). In comparison, patients with a GFR less than 3 mL/min displayed a mean weekly phosphate removal comparable to that of a single HD session (952.9 ± 418.8 mg) [20]. Consistently, Wang and colleagues reported that a weekly phosphate excretion in the urine of patients with a daily urine output > 200 mL was between 300 and 1500 mg, comparable to the quantity removed during a single 4 h HD session [21]. Naturally, this was substantially less in those with a urine volume less or equal to 200 mL per day (769 ± 318 vs. 122 ± 106 mg/week, *p* < 0.001) and associated with a lessened need for phosphate-binding medication [21].

#### 2.1.2. Middle-Molecule Uremic Toxins

Middle-molecule uremic toxins are solutes with molecular weights between 0.5 and 58 kDa, divided into the small middle (0.5–15 kDa), medium middle (15–25 kDa), and large middle (25–58 kDa) categories [22]. This classification, which aligns with the upper limit of glomerular filtration, includes beta 2 microglobulin (B2MG), IL-6, TNF-alpha, various free light chains, and α1-microglobulin, among others. These molecules play significant roles in increasing morbidity and mortality among dialysis patients due to their involvement in inflammatory responses, the cardiovascular system, and other metabolic disturbances. The hemodialysis clearance of middle-molecule uremic toxins can be enhanced with the use of high-flux dialyzers and hemodiafiltration. The HEMO Study showed that the high-flux dialyzer group experienced lower mortality than the low-flux dialyzer group among the subgroups of patients with a dialysis duration of more than 3.7 years [19]. A recent randomized controlled trial demonstrated that the use of high-dose hemodiafiltration resulted in a lower risk of death from any cause than conventional high-flux hemodialysis [23].

Among middle-molecular uremic toxins, B2MG has been the most extensively studied in the literature, showing strong associations with RKF and patient outcomes [24]. In the HEMO post hoc study by Toth-Manikowski et al., pre-HD B2MG levels were 30.7 ± 11.2 mg/L vs. 38.3 ± 14.1 mg/L, respectively (*p* < 0.001) [11]. Furthermore, in a study by Fry et al., patients with a minimal CLurea of <0.5 mL/min exhibited markedly higher B2MG levels in the serum compared to those with even modest CLurea levels of 0.5–1.0 mL/min (28.2 ± 6.2 vs. 23.1 ± 4.6 mg/L, *p* < 0.001) [25].

#### 2.1.3. Protein-Bound Uremic Toxins

P-cresol sulfate (PCS), indoxyl sulfate (IS), and hippurate (HIPP) are representative protein-bound uremic toxins [18,26]. These gut-derived organic anions are mainly eliminated by renal tubular secretion and are much less dialyzable than water-soluble uremic toxins [27,28]. These protein-bound uremic toxin levels are closely correlated with RKF levels [28,29] and have been repeatedly associated with cardiovascular disease and mortality [30,31,32,33,34] with some exceptions [35].

Toth-Manikowski et al. evaluated the renal clearance of PCS, IS, and HIPP in the above-mentioned study using the HEMO Study samples [11] and found that in patients with RKF, when compared with those without, IS and HIPP levels were 11.1% and 23.6% lower, respectively. Each 0.5 mL/min/35 L TBW decrease in CLurea was associated with IS and HIPP levels that were 0.24 mg/dL and 1.10 mg/dL lower, respectively [11]. Interestingly, PCS levels were paradoxically higher among patients with RKF vs. no RKF, but no association was found between CLurea and PCS levels among patients with RKF. Another study evaluated the relative contribution of RKF vs. HD to the clearance of protein-bound uremic toxins, i.e., PCS, ID, and HIPP. With the same combined urea clearance from HD and RKF, patients receiving twice-weekly HD with RKF (CLurea 2.8 ± 1.5 mL/min), compared to those patients receiving thrice-weekly HD without RKF, had lower levels of HIPP (2.7 ± 2.7 mg/dL vs. 5.5 ± 2.6 mg/dL, *p* = 0.02) but similar levels of PCS (4.2 ± 2.1 mg/dL vs. 4.3 ± 1.9 mg/dL, *p* = 1.0) and IS (1.9 ± 0.9 mg/dL vs. 2.3 ± 0.8 mg/dL, *p* = 0.39) [28].

#### 2.1.4. Acidic Metabolites

The contribution of RKF to the control of metabolic acidosis might be one of the reasons for the reported improvement in the nutritional status of patients compared to those lacking this function [36]. Metabolic acidosis typically provokes an intensified catabolic response involving proteins and essential amino acids, ensuring an enhanced branched-chain amino acid (BCAA) catabolism. This, in turn, results in increased protein degradation and decreased protein synthesis. It has further been hypothesized that a better control of plasma BCAA levels may mediate the observed association between RKF and preserved appetite [36]. Thus, the potential of RKF to limit metabolic acidosis may confer a nutritional advantage for patients on chronic dialysis.

### 2.2. Fluid Elimination

Native kidneys play an integral role in maintaining the body’s water balance, largely by regulating the excretion of salt and volume, a mechanism tightly regulated by hormones, including the Renin–Angiotensin–Aldosterone System, Antidiuretic Hormone, and Atrial Natriuretic Peptide [37]. Advanced stages of kidney dysfunction greatly impact this intrinsic kidney capability and can further manifest with an impaired response to drugs that promote water excretion, such as diuretics [38].

Ultrafiltration, or controlled extracorporeal water removal, is used to manage the volume status of patients. The process uses a pressure gradient to remove excess water, with the goal of keeping the patients at their ideal “dry weight”. Given its ability to control the rate and volume of fluid removal, HD allows clinicians to manage fluid overload in patients more effectively than compromised kidneys. While this controlled elimination of excess salt and water allows for short-term control over water and salt balance, if fluid is removed too rapidly [39] or too excessively [40] it also results in an increased risk of adverse clinical outcomes. In the absence of a routinely measured objective marker of fluid overload, a lack of knowledge of a patient’s dry weight accentuates the problem, and a large number of patients undergoing dialysis show fluid overload [41,42].

Numerous cardiovascular advantages have been attributed to the presence of RKF in patients undergoing chronic dialysis. It has been associated with an increased capability for sodium extraction and fluid volume regulation, evidenced by diminished interdialytic weight gain [43]. In a study by Toth-Manikowski et al., patients without RKF, compared with those with an average CLurea of 0.7 ± 0.4 mL/min/35 L and an average urine volume of 1.8 L/week, had 762 mL/week less ultrafiltration when standardized to a weight of 70 kg (*p* < 0.001) [11]. Other studies confirmed that patients with CLurea equal to or greater than 1 mL/min/1.73 m^2^ displayed significantly reduced ultrafiltration requirements [44]. This suggests that the supplemental volume control provided by RKF could potentially account for the inverse relationship established between the extent of RKF and left ventricular hypertrophy, along with systolic dysfunction [45]. Notably, this association persists irrespective of blood pressure or anemia levels [46]. Furthermore, RKF has been linked to decreased blood concentrations of B-type natriuretic peptide and total homocysteine [45].

### 2.3. Immune Function

While the kidneys are not categorized as an immune organ (in contrast to the thymus, bone marrow, spleen, and lymph nodes, which are central to the generation and maturation of immunological cells), they are nevertheless important to maintaining the proper functionality of the immune system. This importance extends beyond their fundamental responsibility of facilitating an internal environmental equilibrium.

The tubulointerstitial compartment, encompassing macrophages and dendritic cells, fulfills a critical role in renal physiology [47,48]. Functioning as vigilant sentinels, these cells continually survey the local milieu for indicators of potential disruption, such as tissue damage or invasive pathogens. Depending upon the circumstance, they may either orchestrate a response to incipient kidney disease or mediate the modulation of established renal pathology [49,50]. Tubular epithelial cells and interstitial cells produce various cytokines and chemokines that regulate immune responses [51]. Cytokines synthesized in the kidneys, such as IL-6 and TNF-α, can modulate inflammation and immune cell activation [52,53].

The kidneys participate in the regulation of autoimmunity by eliminating self-reactive immune cells and promoting the production of regulatory T cells which suppress excessive immune responses [54,55]. Finally, the kidneys are responsible for clearing immune complexes. Experimental studies described a tissue-specific anatomical and functional unit, formed by resident macrophages and peritubular capillary endothelial cells, which monitors the transport of proteins and particles ranging from 20 to 700 kDa or 10 to 200 nm into the kidney interstitium. Kidney-resident macrophages can immediately detect potential infectious particles and immune complexes and initiate an immune response [56]. In contrast, HD does not have the ability to perform antigen presentation, does not replicate the normal production of cytokines and chemokines, and does not effectively clear immune complexes, all of which can contribute to the prevalence of altered immune function, increased inflammation, and susceptibility to infections in individuals undergoing HD [57,58].

The role that kidneys play in immune function may provide an explanation as to why patients on chronic HD exhibiting RKF exhibit lower inflammation levels. An increased inflammatory response, as indicated by C-reactive protein (CRP) levels, has been associated with the loss of RKF [59]. A compelling cross-sectional observational study by de Sequera et al. revealed a correlational relationship between higher RKF and lower inflammation markers [60]. Patients with CLurea > 1 mL/min and urine output > 100 mL/day were found to have a reduced percentage of CD14+/CD16++ inflammatory monocytes (14.6% vs. 28.3%, *p* = 0.02) and lower CRP concentrations (6.2 vs. 21.4 mg/L, *p* = 0.038) [60]. The activation of CD16+ monocytes in patients with low RKF may contribute to endothelial damage, which can potentially precipitate the onset of atherosclerosis [61]. Shafi et al. also reported a similar relationship between RKF and lower levels of inflammatory markers such as CRP and IL-6 [62]. Aligned with these findings, Yang et al. noted that higher urine output in patients on chronic HD correlated with lower levels of high-sensitivity CRP [63].

### 2.4. Lipid Regulation

An important role in lipid metabolism is played by the native kidneys, which regulate the synthesis, transport, and breakdown of lipids in the body [64] and participate in the clearance of lipoproteins and cholesterol from the blood [65]. A decline in native kidney function and the absence of metabolic functions in artificial kidneys contribute to altered lipid metabolism in patients with chronic kidney disease with or without dialysis, manifesting through increased triglyceride levels, decreased high-density lipoprotein cholesterol levels, and altered low-density lipoprotein particle size and composition [66,67,68]. In addition to decreased lipid clearance and altered lipid metabolism, these lipid abnormalities are influenced by inflammation and hormonal imbalances, both of which are more pronounced in individuals undergoing HD.

The role of the kidneys in lipid metabolism might account for the observed differences in lean body mass [36] and lower risk of atherosclerosis [69] and vascular calcifications [70] in patients on chronic HD who have RKF. Compared to patients without RKF, those with RKF have higher fat-free mass index (17.2 ± 1.8 vs. 15.9 ± 1.3 kg/m^2^, *p* = 0.05) [36]. Univariate and multivariate analyses identified higher levels of RKF as a predictive factor for a reduced risk of atherosclerosis (odds ratio 0.95; 95% CI 0.54 to 0.99, *p* = 0.041) [69]. Furthermore, the absence of RKF has been associated with higher abdominal aortic calcification scores, where RKF absence showed an estimate of 0.22 (95% CI 0.08–0.53, *p* = 0.01) in a multivariable linear regression model. Notably, this association was robust against the inclusion of additional vascular calcification predictors such as age, duration of HD treatment, diabetes, CRP, and calcium–phosphorus product [70].

### 2.5. Glucose Regulation

The native kidneys regulate glucose homeostasis through glucose filtration; the reabsorption, degradation, and removal of insulin from the body; and gluconeogenesis [71]. Naturally, HD does not provide metabolic functions such as gluconeogenesis. Glucose, however, is a component of the dialysate solution that helps maintain stable glucose levels in the bloodstream throughout HD treatments [72,73]. Empirical evidence indicated that, compared with the use of a dialysate glucose concentration of 100 mg/dL, a dialysate glucose concentration of 200 mg/dL was associated with heightened vagal tone [74] and more pronounced postdialytic fatigue in diabetic subjects [75].

On the other hand, insulin is adsorbed and removed by the dialyzer; therefore, the concentration of plasma insulin is decreased after HD [76,77,78]. A rapid drop in plasma glucose levels due to HD can lead to a stimulated secretion of counter-regulatory hormones such as glucagon, growth hormone, and adrenocorticotropic hormone. These factors can trigger an elevation in plasma glucose levels after HD. This phenomenon has been called “HD-associated hyperglycemia” [79,80]. So far, studies on the effects of RKF on the glucose metabolism, rates of hypo- or hyperglycemia, and insulin levels of patients on chronic HD are lacking.

### 2.6. Protein Metabolism

The kidneys hold a critical role in protein metabolism by regulating the plasma concentrations of most amino acids and being responsible for the ultimate catabolism of nearly all filtered and secreted proteins. Through a sophisticated glomerular filtration and tubular reabsorption process, the kidneys prevent the loss of vital proteins in urine [81]. Additionally, the kidneys contribute to the de novo synthesis of various critical substances like glucose and amino acids; in particular, arginine and hydroxyproline [82].

Notwithstanding the technologically advanced design of dialyzer membranes allowing them to target the selective diffusion of small-to-medium-sized waste molecules while restricting the passage of larger molecules like proteins, the loss of amino acids during HD can occur [83]. Research has quantified the intradialytic losses of amino acids, estimating a range of 4 to 13 g per dialysis session, which may cause a reduction in plasma amino acid concentrations [84]. It has been speculated that the loss of amino acids through HD could contribute to the genesis of protein–energy wasting and\or the perpetuation of a chronic inflammatory state [85].

The involvement of native kidneys in protein metabolism may underlie the observation that patients on chronic HD who have RKF have better nutritional status [36]. If the production of arginine, an essential amino acid, is maintained to some degree with the RKF, the elevated arginine levels may be transported to the skeletal muscle, consequently boosting protein synthesis [81]. Moreover, the kidneys are vital for the generation of carnitine and leucine keto-acids, thus potentially conferring specific nutritional benefits [86]. In a retrospective study that involved 650 patients initiating chronic HD treatment, those with CLurea > 1 mL/min/1.73 m^2^ demonstrated increased serum albumin concentrations and normalized protein catabolic rate (nPCR) for a span of up to 36 months, as compared to patients with lower RKF levels [44]. Substantiating these findings, a cross-sectional multicenter investigation conducted over 704 patients on chronic HD in Taipei found that every additional liter recorded in the residual 24 h urine volume corresponded to a 1.4 g/L surge in serum albumin [63].

### 2.7. Hormone Production

The kidneys produce several essential hormones, including Erythropoietin (EPO), to regulate the production of red blood cells by the bone marrow; renin, to regulate the body’s salt and water balance and blood pressure; and calcitriol, to regulate calcium homeostasis and bone health. To compensate for the hormonal production deficit associated with advanced kidney dysfunction, which cannot be fulfilled by HD, medications have been developed that emulate the effects of EPO, i.e., erythropoietin-stimulating agents (ESAs) and active vitamin D analogs (VDAs) [87,88].

While ESAs and VDAs are effective biopharmaceutical medications used to treat anemia and secondary hyperparathyroidism, individual responses to these medications can vary based on factors such as concurrent medical conditions, nutritional status, and inflammatory state [89]. In addition, the use of ESAs does carry some potential risks, such as anemia overcorrection, thromboembolic disease, the exacerbation of hypertension, the aggravation of an underlying malignancy, and a heightened incidence of stroke [90].

Ongoing endogenous hormone production in patients with RKF could underlie the association between RKF and improved anemia control in patients on HD [91]. A faster RKF decline during the first year of dialysis has also been associated with ESA hyporesponsiveness and low hemoglobin levels among patients with new-onset chronic HD [92]. Vilar et al. found a reduced weekly ESA dose and reduced ESA resistance index for up to 48 months after HD initiation in patients with CLurea ≥ 1 mL/min/1.73 m^2^, although no significant difference in serum hemoglobin was noted [44]. The CHOICE study also showed that patients with a daily urine output > 250 mL at 1 year after commencing HD required a lower dose of ESA compared with those without (*p* = 0.001). Similar trends were noted with the ESA resistance index [62]. The role of RKF in the transformation of vitamin D into its active form may limit the escalation of parathyroid hormone. This restraint could potentially contribute to beneficial anabolic effects by curbing amino acid liberation from muscle tissue. Consequently, this biochemical process might contribute to the improvements in lean body mass and protein metabolism previously noted in patients with on chronic HD with RKF [36].

## 3. RKF Indices and Their Limitations

To quantify RKF in patients on HD, the indices that are most commonly employed are GFR, CLurea (or Kru), and urine volume.

### 3.1. Glomerular Filtration Rate (GFR)

GFR indexed to body surface area (BSA) serves as a commonly used metric for assessing kidney function in pre-dialysis chronic kidney disease (CKD), widely employed for CKD staging and risk assessments of clinical outcomes. In clinical practice, this is commonly estimated using an equation including demographic and laboratory variables [93]. However, its applicability encounters limitations within the dialysis population: First, GFR solely reflects renal filtration function and does not encompass other essential kidney functions like tubular secretion [27,94]. This was confirmed in a previous study that revealed an association between the kidney clearances of secretory solutes and patient-reported symptoms related to uremia and heart failure [95]. Secondly, the common practice of indexing GFR to BSA may prove inappropriate for patients at extremes of weight, whether underweight or obese, which are highly prevalent in patients on dialysis and often associated with the underlying cause of renal failure [96]. While BSA indexing aims to standardize metabolic waste exposure across diverse body sizes, further research is required to determine its suitability for individuals with extreme body sizes, where BSA markedly deviates from the standard normalized value of 1.73 m^2^. Lastly, given the dearth of evidence, there is concern regarding the practicality of measuring GFR in dialysis populations due to its unclear relationship with more established markers of kidney function such as CLurea, as discussed below. Consequently, the current guidelines for HD patients do not provide any specific recommendations on GFR measurement.

### 3.2. CLurea

CLurea is the most commonly used RKF index in the dialysis population, despite being approximately 20% lower than GFR due to tubular reabsorption [97,98]. Its widespread adoption is attributed to its convertibility into Kt/Vurea, the traditional metric for assessing dialysis adequacy. However, at the time of the writing of this review, the Centers for Medicare & Medicaid Services (CMS) exclusively use dialysis spKt/Vurea as a clinical performance measure for patients undergoing thrice-weekly HD [99].

An inherent limitation of the use of CLurea is the validity of urea as a marker of solute clearance, akin to Kt/Vurea. The diverse range of uremic toxins, including but not limited to B2MG, PCS, and IS, possess kinetic properties distinct from urea—such as molecular weight, hydrophilicity vs. hydrophobicity, volume distribution, electrical charges, and protein binding ratio. Relying on the clearance of a single small solute fails to encompass the combined effects of RKF and dialysis therapy [22]. Furthermore, an ongoing debate revolves around whether clearance measures in the dialysis population should be indexed by TBW, BSA, or other metrics of body size, such as height [100,101].

### 3.3. Urine Volume

Although the urine volume does not directly provide information about kidney solute clearance, it exhibits a robust correlation with residual kidney CLurea in dialysis patients [102,103,104,105]. Beyond its association with kidney function, greater urine volume holds the potential for additional clinical advantages, contributing to improved volume control and potentially leading to enhanced patient-centered outcomes—such as reduced fatigue after HD and a more flexible diet.

It is worth noting that urine volume distinguishes itself from other RKF indices due to its modifiability through diuretic use. In a prospective international cohort of HD patients, diuretic use was linked to reduced interdialytic weight gain, lower odds of hyperkalemia, and decreased cardiac-specific mortality [106]. Moreover, in a separate prospective cohort study involving Korean HD patients, urine volume, as opposed to measured or estimated GFR, was independently correlated with all-cause mortality [107]. Further studies are required to assess whether urine volume offers additional predictive value for outcomes or clinical benefits beyond what is provided by kidney CLurea.

## 4. RKF Evaluation

The quantification of residual kidney function (RKF) primarily relies on timed urine collection methodologies. Creatinine and urea can be used as endogenous filtration markers for the quantification of RKF. The plasma levels of both urea and creatinine increase during the interdialytic period, and hence, their time-averaged concentrations (TACs) in plasma during the urine collection period needs to be estimated to accurately calculate RKF.

The methodologies available to quantify RKF are outlined below and summarized in Table 2 and Table 3.

### 4.1. Timed Urine Collection for GFR

In a pioneering study by Multinovic et al., 38 patients on chronic three-times-weekly HD performed 24 h urine collection during a 24 h interdialytic period; the authors reported two important findings [98]. First, the plasma urea and creatinine levels at 12 h of urine collection was very close to the average of the plasma levels at the start and end of timed urine collection, suggesting that these levels increase linearly during the 24 h following HD, which further indicates that the TAC of plasma urea and creatinine can be estimated as the average of the pre- and post-collection levels. Second, the average of CLurea and creatinine clearance (CLcr) agreed well with the GFR measured by urinary inulin clearance. This was explained by the fact that while CLcr tends to overestimate GFR due to tubular creatinine secretion and CLurea underestimates GFR due to the tubular reabsorption of urea, estimating as an average offsets these two contrasting effects on GFR estimation. However, it should be noted that their study participants had low levels of RKF (only 2 out of 38 patients had CLurea > 2.0 mL/min), and urine was collected during a shorter interdialytic period than the usually recommended ones (24 h vs. 44 or 68 h). Therefore, their findings may not be generalizable to urine collection during interdialytic periods in patients with higher levels of RKF, where the rate of increase in plasma urea and creatinine levels tends to be comparably moderate over time. In the latter scenarios, RKF could be overestimated if based solely on the average of pre- and post-collection plasma solute levels, as the TAC might be higher than this average.

Van Olden et al. studied serial urine collections over a 3-day interdialytic interval among 11 patients undergoing twice-weekly HD, of which 6 had a urine volume greater than 1500 mL during the collection interval [113]. The investigators collected urine samples at each midnight during the 3-day period and compared the kidney clearances of inulin, urea, and creatinine. They observed that the overestimation of GFR by CLcr was at its minimum on the final day of the interdialytic interval (i.e., 0.26 ± 0.60 without cimetidine and 0.10 ± 0.67 with cimetidine), with this last interval averaging approximately 10 h. Based on these findings, they advocated for the utilization of CLcr derived from 8-to-12 h urine collections using pre-HD plasma creatinine as a practical approach for GFR measurement. The researchers argued that employing pre-HD plasma creatinine alone, rather than the average of the pre- and post-urine collection period, would be more suitable, as it would prevent the overestimation of the TAC. This adjustment helps counteract the GFR overestimation caused by tubular creatinine secretion. Notably, the study did not include a comparison between inulin clearance and the averages of kidney CLurea and CLcr.

### 4.2. Timed Urine Collection for CLurea

The major purpose of obtaining CLurea is to assess the combined small-solute clearance adequacy from dialysis and the native kidneys. Here, it should be noted that the traditional dialysis dose measurement Kt/Vurea is scaled to total body water, which is the approximation of urea distribution. Therefore, the kidney CLurea needs to be the plasma *water* clearance, which is approximately 93% of the plasma clearance value assuming normal total protein levels [114]. Alternatively, plasma *water* levels can be estimated using Colton’s formula, which applies a factor of 1 − 0.0107 × total protein (g/dL) to plasma levels [115].

Although TACs in plasma can be estimated as the average of pre- and post-urine collection levels, two- or three-day urine collections and post- and pre-HD blood draws require considerable effort from patients, making them challenging in routine clinical practice. Traditionally, TACs in plasma *water* are estimated by applying a correction factor of 0.9 to pre-HD serum urea nitrogen (SUN). This traditional approach is effective for the typical urine collection protocol with a standard HD dose (i.e., 24 h urine collection before HD over a 2-day interdialytic period while receiving HD with a urea reduction ratio of 65–70%). However, the ratio of TAC SUN to pre-HD SUN is influenced by variables such as dialysis dose, interdialytic interval, and urine collection period. Therefore, the plasma water of CLurea can be more accurately estimated by applying a correction factor (R) based on the following formula [116]:

R = 1.075 − (0.0038 × urea reduction ratio (%) + 0.059) × urine collection period (min)/interdialytic interval (min)

Here, urine collection is supposed to be completed one hour before the subsequent HD session. This equation demonstrated similar CLurea levels to those obtained from formal urea kinetic modeling, with only approximately 5% overestimation among patients undergoing less frequent HD (i.e., once- or twice-weekly HD). Subsequently, CLurea can be converted to weekly or standard kidney Kt/Vurea using the following equations [117,118]:

Weekly or standard kidney Kt/Vurea = {CLurea (mL/min) × 1440 (mins) × 7 (days)}/{Adjusted total body water by Watson’s formula (L) × 1000}

However, the above study conducted by Van Olden et al. revealed that both GFR and CLurea increase during interdialytic intervals, especially after 32 h post-HD or the first day of the interdialytic period [113]. The mean kidney CLurea among the study participants was 1.4–1.5 mL/min during the first 32 h. It then increased to 1.7 mL/min during the second day of the interdialytic period, and further rose to 2.0 mL/min over the average 10 h period preceding the subsequent HD session. This finding suggests that some urine collection protocols might not yield CLurea values that accurately reflect weekly kidney CLurea. In a reanalysis of the original data from Van Olden et al., Daugirdas showed that no further correction was necessary for 24 h urine collections during a 2-day interdialytic interval among patients undergoing three-times-weekly HD [119]. However, for other collection durations (i.e., 12 h or 48 h), the interdialytic intervals (i.e., three or four days) and dialysis frequency (i.e., twice-weekly HD) correction factors to convert the measured CLurea to weekly CLurea were estimated to be either <0.9 or >1.1. For example, a correction factor of 0.89 is necessary for the conversion to weekly kidney CLurea for a 24 h urine collection during a 3-day interdialytic interval among patients on twice-weekly HD [119].

#### Limitations of Timed Urine Collection

Urine collection for the evaluation of RKF in patients on HD faces many challenges that can impact its accuracy. These include the urine collection process itself, the timing required for blood sampling to measure plasma urea and creatinine levels, and the overall duration of urine collection. However, there is no gold-standard method to definitively determine the accuracy of a timed urine collection to this date. In patients not on HD, the measurement of creatinine in a timed urine sample is used as a metric to determine the accuracy of timed urine collection and is motivated by its theoretically stable production from creatine in skeletal muscle and its nearly exclusive elimination by the kidneys [120]. Under steady-state conditions, creatinine excretion in the urine should equal its production, which can be estimated from body size. Patients treated with intermittent HD, however, are typically not in a steady state when timed urine collection is conducted.

Common errors in urine collection involve starting the collection at an incorrect time, accidentally flushing away a urine sample, and failing to bring the collection bottle when leaving home. Urine collection typically spans a 24 h period, and such mistakes are particularly frequent during the daytime when patients are engaged in daily activities, resulting in distraction or forgetfulness. To mitigate these issues, a 12 h urine collection period can alternatively be used, where the collection begins the evening before the dialysis clinic visit. This strategy aims to minimize conflicts with patients’ daily routines, potentially reducing sources of error. This modified approach, supported by a study by Van Olden et al. [108], can simplify the process for patients, encouraging adherence to the protocol and improving the accuracy and reliability of urine collection studies.

### 4.3. GFR Measurement Using Exogenous Filtration Markers

The gold standard of GFR measurement is the kidney clearance of inulin, which requires a continuous infusion of inulin, bladder catheterization, and timed serum and urine collections. GFR can also be measured by using other exogenous filtration markers such as 51Cr-EDTA, 99Tc-DTPA, iothalamate, or iohexol, with the latter two being widely favored in Europe and the USA primarily for practical considerations [121]. While this review does not cover the detailed protocols, they can be found elsewhere [97,122,123,124,125,126].

In pre-dialysis CKD patients, plasma-based clearance methods are generally preferred over kidney clearance methods from a logistics standpoint because the assessment requires only serial plasma sample collections, but no urine sample collections, after a one-time subcutaneous injection of a measured amount of either marker [125]. However, plasma clearance has been found to be higher than urinary clearance by 2 to 5 mL/min per 1.73 m^2^ for iohexol among patients on dialysis [122,123], which is attributed to extra-renal clearance and/or errors in the modeling analysis of the plasma decay curve [127]. A similar finding was also reported with iothalamate [128]. Additionally, plasma clearance methods overestimate GFR by 2 to 13 mL/min per 1.73 m^2^ among patients with significant edema [120], likely because the distribution of the marker takes several days in a setting of expanded extracellular volume [122]. Such estimation errors are very large in proportion to such low levels of kidney function observed among dialysis patients, and hence, urinary clearance methods are preferred over plasma clearance methods for GFR measurement in this population. Although iothalamate can also be secreted into the tubular secretion to some extent [129,130], urinary iothalamate clearance was found to be fairly close to urinary inulin clearance [97]. Nevertheless, the cost, time, and labor required for these assessments remain a barrier for many centers [121].

### 4.4. RKF Estimating Equations without Timed Urine Collection

Interest in exploring endogenous markers to estimate GFR and kidney CLurea, without the necessity for timed urine collection, is ongoing. Several equations have been developed based on serum markers including creatinine, B2MG, cystatin C, and beta-trace protein (Table 2 and Table 3) [108,109,110,111,112,131]. However, all equations published so far have shown suboptimal precision for clinical applications. The proportion of estimates within an error range of 2 mL/min/1.73 m^2^ has been arbitrarily proposed and used for assessing accuracy, but such an error is practically too large in the dialysis population. Some studies lacked external validation, rendering their generalizability unclear [108,109,110,131]. Additionally, the considerable inter-assay variability for B2MG and beta-trace protein limit the widespread use of these equations [126].

## 5. Potential Benefits of Targeted Interventions Aimed at Preserving RKF

Lower RKF levels and RKF decline among dialysis patients have been independently associated with higher mortality, greater morbidity burden, and poorer quality of life, and hence preserving RKF potentially leads to better clinical outcomes [132,133,134]. Additionally, HD prescriptions can be individualized based on a patient’s RKF, and less frequent HD can be considered among patients with substantial RKF while taking account of comorbidities and specific needs [135,136].

Potential strategies for preserving RKF include promoting adequate blood pressure control while preventing intradialytic hypotension, avoiding nephrotoxic agents, starting with less frequent HD at dialysis initiation, implementing a low-protein diet, and using biocompatible dialysis membranes and ultrapure dialysate, as discussed in detail elsewhere [132,137]. Sodium–glucose cotransporter-2 inhibitors have been shown to slow the progression of chronic kidney disease [138], and therefore may provide protection against RKF among dialysis patients [139]. Conversely, frequent HD may accelerate RKF loss and increase mortality risk among patients with preserved RKF [140,141]. It should be noted that the goal of preserving RKF should not compromise other aspects of patient care; patients must not be kept in a state of volume overload [142], and the use of renin–angiotensin system inhibitors should be maintained unless contraindicated [132,137].

## 6. Conclusions

The contribution of RKF extends beyond the removal of uremic solutes, with the consequences of RKF loss affecting immune, metabolic, and hormonal regulation. To recognize a few shortcomings in the dialysis field, the evaluation of RKF is limited to the removal of a few uremic toxins, and dialysis treatments are far from being so-called “kidney replacement therapies”. More innovative research is needed to narrow the effectiveness gap between native kidney function and kidney dialysis therapies.

## Figures and Tables

**Table 1:** Comparison between the functions of natural kidneys and the dialyzer.

Function	Natural Kidney	Artificial Kidney	Advantages Conferred by Residual Kidney Function
Solute and acid–base homeostasis	Clearance of an entire array of solutes, from small- to large- and protein- bound molecules. Excretion of hydrogen ions and generation of bicarbonate ions.	Clearance of small- and middle-molecular-weight solutes. Removal of acid and addition of buffer base.	Lower concentration of middle- and protein-bound molecules. Lower phosphate levels.
Fluid balance	Adjusts the volume and the concentration of the urine to maintain the balance of water.	Controlled removal of water through ultrafiltration.	Lower interdialytic weight gain.
Immune function	Antigen presentation. Cytokine and chemokine production. Regulation of autoimmunity. Clearance of immune complexes.	Aids immune function through clearance of uremic toxins.	Lower inflammation.
Lipid regulation	Clearance of lipoproteins and cholesterol.	None.	Better lean body mass. Lower risk of atherosclerosis.Lower vascular calcification burden.
Glucose homeostasis	Glucose filtration and reabsorption. Regulation of insulin. Gluconeogenesis.	Dialysate with added glucose. Removes insulin.	Better nutritional status.
Protein metabolism	Regulates the amino acid plasma concentration.	Removes amino acids.	Better nutritional status.
Endocrine function	Erythropoietin. 1,25-dihydroxy vitamin D.	Necessitates the addition of pharmacologic products: erythropoietin-stimulating agents (ESAs), active vitamin D analogs.	Lower ESA requirements. Better anemia control.

**Table 2:** GFR estimation equations using endogenous filtration markers.

**Hoek, F.J. et al.** [108]	**Internal validation only**
Equation	GFR (mL/min per 1.73 m^2^) = −0.77 + {21/Cystatin C (mg/L)} *
- Performance indices	Systemic bias 0.24 (SD, 1.24), 95% limits of agreement (−2.2, 2.68), r = 0.48
**Vilar, E. et al.** [109]	**Internal validation only**
Equation (1)	GFR (mL/min) = (160.3/B2MG) − 4.2
- Performance indices	Systemic bias 1.4 (SD, 1.92), 95% limits of agreement (−2.35, 5.16), r ^ 2 = 0.55
Equation (2)	GFR (mL/min per 1.73 m^2^) = {142.2/B2MD (mg/L)} + {899.8/Creatinine (μmol/L)} + 0.0.13 × Pre-HD Weight (kg) − 5.63
- Performance indices	Not available
**Wong, J. et al.** [110]	**Internal validation only**
Equation	GFR (mL/min) = {13.471/BTP (mg/L)} + {52.379/B2MG (mg/L)} + {782.909/Creatinine (μmol/L)} − 3.939 + 0.519 (if male)
- Performance indices	Systemic bias −0.64 (95%CI, −0.89 to −0.39), 95% limits of agreement (−2.84, 1.57), r = 0.783
**Shafi, T. et al.** [111]	**External validation**
Equation (1)	GFR (mL/min per 1.73 m^2^) = 2852 × B2MG (mg/L) ^ (2.417) × 1.592 if male **
- Performance indices	Systemic bias 1.0 (95%CI, 0.9 to 1.1), interquartile range of bias 1.9 (95%Ci, 1.7 to 2.1)
Equation (2)	GFR (mL/min per 1.73 m^2^) = 673 × BTP (mg/L) ^ (−1.406) × B2MG (mg/L) ^ (−1.096) × 1.670 if male **
- Performance indices	Systemic bias 0.7 (95%CI, 0.6 to 0.8), IQR of bias 1.8 (95%Ci, 1.6 to 1.9)
**Steubl, D. et al.** [112]	**External validation**
Equation (1)	GFR (mL/min per 1.73 m^2^) = 39 × {B2MG (mg/L)/23} ^ (0.144) × Creatinine (mg/dL) ^ (−1.152) [For B2M ≤ 23 mg/L]
	GFR (mL/min per 1.73 m^2^) = 39 × {B2MG (mg/L)/23} ^ (−2.129) × Creatinine (mg/dL) ^ (−1.152) [For B2M > 23 mg/L]
- Performance indices	Systemic bias 0.4 (95%CI, 0.4 to 0.5), IQR of bias 1.8 (95%CI, 1.6 to 2.0)
Equation (2)	GFR (mL/min per 1.73 m^2^) = 32 × BTP (mg/L) ^ (−1.126) × {B2MG (mg/L)/23} ^ (0.271) [For B2M ≤ 23 mg/L]
	GFR (mL/min per 1.73 m^2^) = 32 × BTP (mg/L) ^ (−1.126) × {B2MG (mg/L)/23} ^ (−2.133) [For B2M > 23 mg/L]
- Performance indices	Systemic bias 0.1 (95%CI, 0.0 to 0.3), IQR of bias 1.8 (95%CI, 1.6 to 2.0)

* Negative proportional bias; ** J-shaped proportional bias; abbreviations: B2M, β2-microglobulin; BTP, β-trace-protein; IQR, interquartile range.

**Table 3:** Kidney urea clearance (CLurea) estimation equations using endogenous filtration markers.

**Wong, J. et al.** [110]	**Internal validation only**
Equation	CLurea (mL/min) = {90.97/BTP (mg/L)} + {37.568/B2MG (mg/L)} − 2.049 + 0.402 (if Caucasian)
- Perfomance indices	Systemic bias −0.50 (95%CI, −0.25 to −0.75), 95% limits of agreement (−2.03, 1.04), r = 0.762
**Shafi, T. et al.** [111]	**External validation**
Equation (1)	CLurea (mL/min) = 2852 × B2MG (mg/L) ^ (2.417) × 1.592 if male *
- Perfomance indices	Systemic bias 0.7 (95%CI, 0.6 to 0.8), IQR of bias 1.6 (95%CI, 1.5 to 1.7)
Equation (2)	CLurea (mL/min) = 673 × BTP (mg/L) ^ (−1.406) × B2MG (mg/L) ^ (−1.096) × 1.670 if male *
- Perfomance indices	Systemic bias 0.5 (95%CI, 0.4 to 0.6), IQR of bias 1.5 (95%CI, 1.4 to 1.7)
**Steubl, D. et al.** [112]	**External validation**
Equation (1)	CLurea (mL/min) = 2 × {B2MG (mg/L)/24} ^ (−0.678) [For B2M < 24 mg/L]
	CLurea (mL/min) = 2 × {B2MG (mg/L)/24} ^ (−2.880) [For B2M > 24 mg/L]
- Perfomance indices	Systemic bias 0.6 (95%CI, 0.6 to 0.7), IQR of bias 1.5 (95%CI, 1.4 to 1.7)
Equation (2)	CLurea (mL/min) = 16 × BTP (mg/L) ^ (−1.02) × {B2MG (mg/L)/24} ^ (0.159) [For B2M < 24 mg/L]
	CLurea (mL/min) = 16 × BTP (mg/L) ^ (−1.02) × {B2MG (mg/L)/24} ^ (−2.187) [For B2M > 24 mg/L]
- Perfomance indices	Systemic bias 0.4 (95%CI, 0.3 to 0.5), IQR of bias 1.5 (95%CI, 1.3 to 1.6)

* Proportional bias with a J-shape association; abbreviations: B2MG, beta 2 microglobulin; BTP, β-trace protein; IQR, interquartile range.

## Data Availability

Not applicable.

## References

[1] Kong J., Davies M., Mount P. (2018). The importance of residual kidney function in haemodialysis patients. Nephrology.

[2] Alrowiyti I.M., Bargman J. (2023). A Review of Residual Kidney Function in Peritoneal Dialysis Patients. Indian J. Nephrol..

[3] Murea M., Deira J., Kalantar-Zadeh K., Casino F.G., Basile C. (2022). The spectrum of kidney dysfunction requiring chronic dialysis therapy: Implications for clinical practice and future clinical trials. Semin. Dial..

[4] Said N., Lau W.J., Ho Y.C., Lim S.K., Zainol Abidin M.N., Ismail A.F. (2021). A Review of Commercial Developments and Recent Laboratory Research of Dialyzers and Membranes for Hemodialysis Application. Membranes.

[5] Murea M., Flythe J.E., Anjay R., Emaad A.M., Gupta N., Kovach C., Vachharajani T.J., Kalantar-Zadeh K., Casino F.G., Basile C. (2022). Kidney dysfunction requiring dialysis is a heterogeneous syndrome: We should treat it like one. Curr. Opin. Nephrol. Hypertens..

[6] Meyer T.W., Hostetter T.H. (2007). Uremia. N. Engl. J. Med..

[7] Glorieux G., Tattersall J. (2015). Uraemic toxins and new methods to control their accumulation: Game changers for the concept of dialysis adequacy. Clin. Kidney J..

[8] Bello-Reuss E., Reuss L., Klahr S. (1983). Homeostatic and Excretory Functions of the Kidney. The Kidney and Body Fluids in Health and Disease.

[9] Meyer T.W., Sirich T.L., Hostetter T.H. (2011). Dialysis cannot be dosed. Semin. Dial..

[10] Bohle A., Aeikens B., Eenboom A., Fronholt L., Plate W.R., Xiao J.C., Greschniok A., Wehrmann M. (1998). Human glomerular structure under normal conditions and in isolated glomerular disease. Kidney Int. Suppl..

[11] Toth-Manikowski S.M., Sirich T.L., Meyer T.W., Hostetter T.H., Hwang S., Plummer N.S., Hai X., Coresh J., Powe N.R., Shafi T. (2020). Contribution of ‘clinically negligible’ residual kidney function to clearance of uremic solutes. Nephrol. Dial. Transplant..

[12] Suchy-Dicey A.M., Laha T., Hoofnagle A., Newitt R., Sirich T.L., Meyer T.W., Thummel K.E., Yanez N.D., Himmelfarb J., Weiss N.S. (2016). Tubular Secretion in CKD. J. Am. Soc. Nephrol..

[13] Mair R.D., Sirich T.L., Meyer T.W. (2018). Uremic Toxin Clearance and Cardiovascular Toxicities. Toxins.

[14] Johnson W.J., Hagge W.W., Wagoner R.D., Dinapoli R.P., Rosevear J.W. (1972). Effects of urea loading in patients with far-advanced renal failure. Mayo Clin. Proc..

[15] Velasquez M.T., Ramezani A., Raj D.S. (2015). Urea and protein carbamylation in ESRD: Surrogate markers or partners in crime?. Kidney Int..

[16] Jaworska K., Hering D., Mosieniak G., Bielak-Zmijewska A., Pilz M., Konwerski M., Gasecka A., Kapłon-Cieślicka A., Filipiak K., Sikora E. (2019). TMA, A Forgotten Uremic Toxin, but Not TMAO, Is Involved in Cardiovascular Pathology. Toxins.

[17] Hsu B.G., Wang C.H., Lin Y.L., Lai Y.H., Tsai J.P. (2022). Serum Trimethylamine N-Oxide Level Is Associated with Peripheral Arterial Stiffness in Advanced Non-Dialysis Chronic Kidney Disease Patients. Toxins.

[18] Poesen R., Claes K., Evenepoel P., de Loor H., Augustijns P., Kuypers D., Meijers B. (2016). Microbiota-Derived Phenylacetylglutamine Associates with Overall Mortality and Cardiovascular Disease in Patients with CKD. J. Am. Soc. Nephrol..

[19] Eknoyan G., Beck G.J., Cheung A.K., Daugirdas J.T., Greene T., Kusek J.W., Allon M., Bailey J., Delmez J.A., Depner T.A. (2002). Effect of dialysis dose and membrane flux in maintenance hemodialysis. N. Engl. J. Med..

[20] Iwasawa H., Nakao T., Matsumoto H., Okada T., Nagaoka Y., Wada T. (2013). Phosphate handling by end-stage kidneys and benefits of residual renal function on phosphate removal in patients on haemodialysis. Nephrology.

[21] Wang M., You L., Li H., Lin Y., Zhang Z., Hao C., Chen J. (2013). Association of circulating fibroblast growth factor-23 with renal phosphate excretion among hemodialysis patients with residual renal function. Clin. J. Am. Soc. Nephrol..

[22] Rosner M.H., Reis T., Husain-Syed F., Vanholder R., Hutchison C., Stenvinkel P., Blankestijn P.J., Cozzolino M., Juillard L., Kashani K. (2021). Classification of Uremic Toxins and Their Role in Kidney Failure. Clin. J. Am. Soc. Nephrol..

[23] Blankestijn P.J., Vernooij R.W.M., Hockham C., Strippoli G.F.M., Canaud B., Hegbrant J., Barth C., Covic A., Cromm K., Cucui A. (2023). Effect of Hemodiafiltration or Hemodialysis on Mortality in Kidney Failure. N. Engl. J. Med..

[24] Cheung A.K., Levin N.W., Greene T., Agodoa L., Bailey J., Beck G., Clark W., Levey A.S., Leypoldt J.K., Ornt D.B. (2003). Effects of high-flux hemodialysis on clinical outcomes: Results of the HEMO study. J. Am. Soc. Nephrol..

[25] Fry A.C., Singh D.K., Chandna S.M., Farrington K. (2007). Relative importance of residual renal function and convection in determining beta-2-microglobulin levels in high-flux haemodialysis and on-line haemodiafiltration. Blood Purif..

[26] Lowenstein J., Grantham J.J. (2017). Residual renal function: A paradigm shift. Kidney Int..

[27] Sirich T.L., Funk B.A., Plummer N.S., Hostetter T.H., Meyer T.W. (2014). Prominent accumulation in hemodialysis patients of solutes normally cleared by tubular secretion. J. Am. Soc. Nephrol..

[28] Marquez I.O., Tambra S., Luo F.Y., Li Y., Plummer N.S., Hostetter T.H., Meyer T.W. (2011). Contribution of residual function to removal of protein-bound solutes in hemodialysis. Clin. J. Am. Soc. Nephrol..

[29] Leong S.C., Sao J.N., Taussig A., Plummer N.S., Meyer T.W., Sirich T.L. (2018). Residual Function Effectively Controls Plasma Concentrations of Secreted Solutes in Patients on Twice Weekly Hemodialysis. J. Am. Soc. Nephrol..

[30] Bammens B., Evenepoel P., Keuleers H., Verbeke K., Vanrenterghem Y. (2006). Free serum concentrations of the protein-bound retention solute p-cresol predict mortality in hemodialysis patients. Kidney Int..

[31] Liabeuf S., Barreto D.V., Barreto F.C., Meert N., Glorieux G., Schepers E., Temmar M., Choukroun G., Vanholder R., Massy Z.A. (2010). Free p-cresylsulphate is a predictor of mortality in patients at different stages of chronic kidney disease. Nephrol. Dial. Transplant..

[32] Barreto F.C., Barreto D.V., Liabeuf S., Meert N., Glorieux G., Temmar M., Choukroun G., Vanholder R., Massy Z.A. (2009). Serum indoxyl sulfate is associated with vascular disease and mortality in chronic kidney disease patients. Clin. J. Am. Soc. Nephrol..

[33] Meijers B.K., Claes K., Bammens B., de Loor H., Viaene L., Verbeke K., Kuypers D., Vanrenterghem Y., Evenepoel P. (2010). p-Cresol and cardiovascular risk in mild-to-moderate kidney disease. Clin. J. Am. Soc. Nephrol..

[34] Meijers B.K., Bammens B., De Moor B., Verbeke K., Vanrenterghem Y., Evenepoel P. (2008). Free p-cresol is associated with cardiovascular disease in hemodialysis patients. Kidney Int..

[35] Shafi T., Sirich T.L., Meyer T.W., Hostetter T.H., Plummer N.S., Hwang S., Melamed M.L., Banerjee T., Coresh J., Powe N.R. (2017). Results of the HEMO Study suggest that p-cresol sulfate and indoxyl sulfate are not associated with cardiovascular outcomes. Kidney Int..

[36] Suda T., Hiroshige K., Ohta T., Watanabe Y., Iwamoto M., Kanegae K., Ohtani A., Nakashima Y. (2000). The contribution of residual renal function to overall nutritional status in chronic haemodialysis patients. Nephrol. Dial. Transplant..

[37] Sterns R.H., Kimmel P.L., Rosenberg M.E. (2020). Chapter 38—Water Homeostasis in Chronic Kidney Disease. Chronic Renal Disease.

[38] Su W., Cao R., Zhang X.Y., Guan Y. (2020). Aquaporins in the kidney: Physiology and pathophysiology. Am. J. Physiol. Renal Physiol..

[39] Assimon M.M., Flythe J.E. (2015). Rapid ultrafiltration rates and outcomes among hemodialysis patients: Re-examining the evidence base. Curr. Opin. Nephrol. Hypertens..

[40] Dekker M.J., Marcelli D., Canaud B.J., Carioni P., Wang Y., Grassmann A., Konings C.J., Kotanko P., Leunissen K.M., Levin N.W. (2017). Impact of fluid status and inflammation and their interaction on survival: A study in an international hemodialysis patient cohort. Kidney Int..

[41] Zoccali C., Moissl U., Chazot C., Mallamaci F., Tripepi G., Arkossy O., Wabel P., Stuard S. (2017). Chronic Fluid Overload and Mortality in ESRD. J. Am. Soc. Nephrol..

[42] Moissl U., Fuentes L.R., Hakim M.I., Hassler M., Kothari D.A., Rosales L., Zhu F., Raimann J.G., Thijssen S., Kotanko P. (2022). Prevalence of fluid overload in an urban US hemodialysis population: A cross-sectional study. Hemodial. Int..

[43] Morduchowicz G., Winkler J., Zabludowski J.R., Boner G. (1994). Effects of residual renal function in haemodialysis patients. Int. Urol. Nephrol..

[44] Vilar E., Wellsted D., Chandna S.M., Greenwood R.N., Farrington K. (2009). Residual renal function improves outcome in incremental haemodialysis despite reduced dialysis dose. Nephrol. Dial. Transplant..

[45] Ma T., Ding G. (2013). Effects of residual renal function on left ventricle and analysis of related factors in patients with hemodialysis. Ren. Fail..

[46] Wang A.Y., Wang M., Woo J., Law M.C., Chow K.M., Li P.K., Lui S.F., Sanderson J.E. (2002). A novel association between residual renal function and left ventricular hypertrophy in peritoneal dialysis patients. Kidney Int..

[47] Kurts C., Ginhoux F., Panzer U. (2020). Kidney dendritic cells: Fundamental biology and functional roles in health and disease. Nat. Rev. Nephrol..

[48] Lin J., Wang H., Liu C., Cheng A., Deng Q., Zhu H., Chen J. (2021). Dendritic Cells: Versatile Players in Renal Transplantation. Front. Immunol..

[49] Rogers N.M., Ferenbach D.A., Isenberg J.S., Thomson A.W., Hughes J. (2014). Dendritic cells and macrophages in the kidney: A spectrum of good and evil. Nat. Rev. Nephrol..

[50] Weisheit C.K., Engel D.R., Kurts C. (2015). Dendritic Cells and Macrophages: Sentinels in the Kidney. Clin. J. Am. Soc. Nephrol..

[51] Jamaly S., Rakaee M., Abdi R., Tsokos G.C., Fenton K.A. (2021). Interplay of immune and kidney resident cells in the formation of tertiary lymphoid structures in lupus nephritis. Autoimmun. Rev..

[52] Leavy O. (2014). Macrophages: Early antifungal defence in kidneys. Nat. Rev. Immunol..

[53] Lionakis M.S., Swamydas M., Fischer B.G., Plantinga T.S., Johnson M.D., Jaeger M., Green N.M., Masedunskas A., Weigert R., Mikelis C. (2013). CX3CR1-dependent renal macrophage survival promotes Candida control and host survival. J. Clin. Investig..

[54] Lukacs-Kornek V., Burgdorf S., Diehl L., Specht S., Kornek M., Kurts C. (2008). The kidney-renal lymph node-system contributes to cross-tolerance against innocuous circulating antigen. J. Immunol..

[55] Teteris S.A., Engel D.R., Kurts C. (2011). Homeostatic and pathogenic role of renal dendritic cells. Kidney Int..

[56] Stamatiades E.G., Tremblay M.E., Bohm M., Crozet L., Bisht K., Kao D., Coelho C., Fan X., Yewdell W.T., Davidson A. (2016). Immune Monitoring of Trans-endothelial Transport by Kidney-Resident Macrophages. Cell.

[57] Cobo G., Lindholm B., Stenvinkel P. (2018). Chronic inflammation in end-stage renal disease and dialysis. Nephrol. Dial. Transplant..

[58] Campo S., Lacquaniti A., Trombetta D., Smeriglio A., Monardo P. (2022). Immune System Dysfunction and Inflammation in Hemodialysis Patients: Two Sides of the Same Coin. J. Clin. Med..

[59] Wang A.Y., Wang M., Woo J., Lam C.W., Lui S.F., Li P.K., Sanderson J.E. (2004). Inflammation, residual kidney function, and cardiac hypertrophy are interrelated and combine adversely to enhance mortality and cardiovascular death risk of peritoneal dialysis patients. J. Am. Soc. Nephrol..

[60] de Sequera P., Corchete E., Bohorquez L., Albalate M., Perez-Garcia R., Alique M., Marques M., García-Menéndez E., Portolés J., Ramirez R. (2017). Residual Renal Function in Hemodialysis and Inflammation. Ther. Apher. Dial..

[61] Wildgruber M., Aschenbrenner T., Wendorff H., Czubba M., Glinzer A., Haller B., Schiemann M., Zimmermann A., Berger H., Eckstein H.H. (2016). The “Intermediate” CD14(++)CD16(+) monocyte subset increases in severe peripheral artery disease in humans. Sci. Rep..

[62] Shafi T., Jaar B.G., Plantinga L.C., Fink N.E., Sadler J.H., Parekh R.S., Powe N.R., Coresh J. (2010). Association of residual urine output with mortality, quality of life, and inflammation in incident hemodialysis patients: The Choices for Healthy Outcomes in Caring for End-Stage Renal Disease (CHOICE) Study. Am. J. Kidney Dis..

[63] Yang P.Y., Lin J.L., Lin-Tan D.T., Hsu C.W., Yen T.H., Chen K.H., Ho T.C. (2009). Residual daily urine volume association with inflammation and nutrition status in maintenance hemodialysis patients. Ren. Fail..

[64] Moestrup S.K., Nielsen L.B. (2005). The role of the kidney in lipid metabolism. Curr. Opin. Lipidol..

[65] Mitrofanova A., Merscher S., Fornoni A. (2023). Kidney lipid dysmetabolism and lipid droplet accumulation in chronic kidney disease. Nat. Rev. Nephrol..

[66] Vaziri N.D. (2006). Dyslipidemia of chronic renal failure: The nature, mechanisms, and potential consequences. Am. J. Physiol. Renal Physiol..

[67] Kaysen G.A. (2006). Dyslipidemia in chronic kidney disease: Causes and consequences. Kidney Int..

[68] Bulbul M.C., Dagel T., Afsar B., Ulusu N.N., Kuwabara M., Covic A., Kanbay M. (2018). Disorders of Lipid Metabolism in Chronic Kidney Disease. Blood Purif..

[69] Rroji M., Spahia N., Seferi S., Barbullushi M., Spasovski G. (2017). Influence of Residual Renal Function in Carotid Modeling as a Marker of Early Atherosclerosis in Dialysis Patients. Ther. Apher. Dial..

[70] Chen H.C., Chou C.Y., Jheng J.S., Chen I.R., Liang C.C., Wang S.M., Liu J.H., Lin S.Y., Kuo H.L., Wang I.K. (2016). Loss of Residual Renal Function is Associated With Vascular Calcification in Hemodialysis Patients. Ther. Apher. Dial..

[71] DeFronzo R.A., Davidson J.A., Del Prato S. (2012). The role of the kidneys in glucose homeostasis: A new path towards normalizing glycaemia. Diabetes Obes. Metab..

[72] Burmeister J.E., Scapini A., da Rosa Miltersteiner D., da Costa M.G., Campos B.M. (2007). Glucose-added dialysis fluid prevents asymptomatic hypoglycaemia in regular haemodialysis. Nephrol. Dial. Transplant..

[73] Abe M., Kaizu K., Matsumoto K. (2007). Plasma insulin is removed by hemodialysis: Evaluation of the relation between plasma insulin and glucose by using a dialysate with or without glucose. Ther. Apher. Dial..

[74] Ferrario M., Raimann J.G., Thijssen S., Signorini M.G., Kruse A., Diaz-Buxo J.A., Cerutti S., Levin N.W., Kotanko P. (2011). Effects of dialysate glucose concentration on heart rate variability in chronic hemodialysis patients: Results of a prospective randomized trial. Kidney Blood Press. Res..

[75] Raimann J.G., Kruse A., Thijssen S., Kuntsevich V., Diaz-Buxo J.A., Levin N.W., Kotanko P. (2010). Fatigue in hemodialysis patients with and without diabetes: Results from a randomized controlled trial of two glucose-containing dialysates. Diabetes Care.

[76] Abe M., Kikuchi F., Kaizu K., Matsumoto K. (2008). The influence of hemodialysis membranes on the plasma insulin level of diabetic patients on maintenance hemodialysis. Clin. Nephrol.

[77] Abe M., Okada K., Matsumoto K. (2008). Plasma insulin and C-peptide concentrations in diabetic patients undergoing hemodialysis: Comparison with five types of high-flux dialyzer membranes. Diabetes Res. Clin. Pract..

[78] Abe M., Okada K., Ikeda K., Matsumoto S., Soma M., Matsumoto K. (2011). Characterization of insulin adsorption behavior of dialyzer membranes used in hemodialysis. Artif. Organs.

[79] Abe M., Kaizu K., Matsumoto K. (2007). Evaluation of the hemodialysis-induced changes in plasma glucose and insulin concentrations in diabetic patients: Comparison between the hemodialysis and non-hemodialysis days. Ther. Apher. Dial..

[80] Hayashi A., Shimizu N., Suzuki A., Matoba K., Momozono A., Masaki T., Ogawa A., Moriguchi I., Takano K., Kobayashi N. (2021). Hemodialysis-Related Glycemic Disarray Proven by Continuous Glucose Monitoring; Glycemic Markers and Hypoglycemia. Diabetes Care.

[81] Morris S.M. (1992). Regulation of enzymes of urea and arginine synthesis. Annu. Rev. Nutr..

[82] Garibotto G., Sofia A., Saffioti S., Bonanni A., Mannucci I., Verzola D. (2010). Amino acid and protein metabolism in the human kidney and in patients with chronic kidney disease. Clin. Nutr..

[83] Prado de Negreiros Nogueira Maduro I., Elias N.M., Nonino Borges C.B., Padovan G.J., Cardeal da Costa J.A., Marchini J.S. (2007). Total nitrogen and free amino acid losses and protein calorie malnutrition of hemodialysis patients: Do they really matter?. Nephron Clin. Pract..

[84] Post A., Kremer D., Groothof D., van der Veen Y., de Blaauw P., van der Krogt J., Kema I.P., Westerhuis R., Heiner-Fokkema M.R., Bakker S.J.L. (2022). Amino Acid Homeostasis and Fatigue in Chronic Hemodialysis Patients. Nutrients.

[85] Hendriks F.K., Smeets J.S.J., Broers N.J.H., van Kranenburg J.M.X., van der Sande F.M., Kooman J.P., van Loon L.J.C. (2020). End-Stage Renal Disease Patients Lose a Substantial Amount of Amino Acids during Hemodialysis. J. Nutr..

[86] Abumrad N.N., Williams P., Frexes-Steed M., Geer R., Flakoll P., Cersosimo E., Brown L.L., Melki I., Bulus N., Hourani H. (1989). Inter-organ metabolism of amino acids in vivo. Diabetes Metab. Rev..

[87] Sahay M., Kalra S., Bandgar T. (2012). Renal endocrinology: The new frontier. Indian J. Endocrinol. Metab..

[88] Acharya V., Olivero J. (2018). The Kidney as an Endocrine Organ. Methodist Debakey Cardiovasc. J..

[89] Sibbel S.P., Koro C.E., Brunelli S.M., Cobitz A.R. (2015). Characterization of chronic and acute ESA hyporesponse: A retrospective cohort study of hemodialysis patients. BMC Nephrol..

[90] Katagiri D. (2014). Benefits and risks of erythrocyte-stimulating agents. World J. Clin. Urol..

[91] Penne E.L., van der Weerd N.C., Grooteman M.P., Mazairac A.H., van den Dorpel M.A., Nube M.J., Bots M.L., Levesque R., ter Wee P.M., Blankestijn P.J. (2011). Role of residual renal function in phosphate control and anemia management in chronic hemodialysis patients. Clin. J. Am. Soc. Nephrol..

[92] Kimura H., Sy J., Okuda Y., Wenziger C., Hanna R., Obi Y., Rhee C.M., Kovesdy C.P., Kalantar-Zadeh K., Streja E. (2021). A faster decline of residual kidney function and erythropoietin stimulating agent hyporesponsiveness in incident hemodialysis patients. Hemodial. Int..

[93] Inker L.A., Eneanya N.D., Coresh J., Tighiouart H., Wang D., Sang Y., Crews D.C., Doria A., Estrella M.M., Froissart M. (2021). New Creatinine- and Cystatin C-Based Equations to Estimate GFR without Race. N. Engl. J. Med..

[94] Hsueh C.H., Yoshida K., Zhao P., Meyer T.W., Zhang L., Huang S.M., Giacomini K.M. (2016). Identification and Quantitative Assessment of Uremic Solutes as Inhibitors of Renal Organic Anion Transporters, OAT1 and OAT3. Mol. Pharm..

[95] Wang K., Nguyen M., Chen Y., Hoofnagle A.N., Becker J.O., Zelnick L.R., Kundzins J., Goodling A., Himmelfarb J., Kestenbaum B. (2020). Association of Tubular Solute Clearance with Symptom Burden in Incident Peritoneal Dialysis. Clin. J. Am. Soc. Nephrol..

[96] Chang A.R., Zafar W., Grams M.E. (2018). Kidney Function in Obesity-Challenges in Indexing and Estimation. Adv. Chronic Kidney Dis..

[97] Milutinovic J., Cutler R.E., Hoover P., Meijsen B., Scribner B.H. (1975). Measurement of residual glomerular filtration rate in the patient receiving repetitive hemodialysis. Kidney Int..

[98] Levey A.S., Bosch J.P., Lewis J.B., Greene T., Rogers N., Roth D. (1999). A more accurate method to estimate glomerular filtration rate from serum creatinine: A new prediction equation. Modification of Diet in Renal Disease Study Group. Ann. Intern. Med..

[99] Griffin S.M., Marr J., Kapke A., Jin Y., Pearson J., Esposito D., Young E.W. (2023). Mortality Risk of Patients Treated in Dialysis Facilities with Payment Reductions under ESRD Quality Incentive Program. Clin. J. Am. Soc. Nephrol..

[100] Daugirdas J.T., Greene T., Chertow G.M., Depner T.A. (2010). Can rescaling dose of dialysis to body surface area in the HEMO study explain the different responses to dose in women versus men?. Clin. J. Am. Soc. Nephrol..

[101] Daugirdas J.T. (2016). You’re Not Big--You’re Just Tall, That’s All!. J. Am. Soc. Nephrol..

[102] Obi Y., Streja E., Rhee C.M., Ravel V., Amin A.N., Cupisti A., Chen J., Mathew A.T., Kovesdy C.P., Mehrotra R. (2016). Incremental Hemodialysis, Residual Kidney Function, and Mortality Risk in Incident Dialysis Patients: A Cohort Study. Am. J. Kidney Dis..

[103] Obi Y., Rhee C.M., Mathew A.T., Shah G., Streja E., Brunelli S.M., Kovesdy C.P., Mehrotra R., Kalantar-Zadeh K. (2016). Residual Kidney Function Decline and Mortality in Incident Hemodialysis Patients. J. Am. Soc. Nephrol..

[104] Chin A.I., Sheth V., Kim J., Bang H. (2019). Estimating Residual Native Kidney Urea Clearance in Hemodialysis Patients with and without 24-Hour Urine Volume. Kidney Med..

[105] Pinto J., Debowska M., Gomez R., Waniewski J., Lindholm B. (2022). Urine volume as an estimator of residual renal clearance and urinary removal of solutes in patients undergoing peritoneal dialysis. Sci. Rep..

[106] Bragg-Gresham J.L., Fissell R.B., Mason N.A., Bailie G.R., Gillespie B.W., Wizemann V., Cruz J.M., Akiba T., Kurokawa K., Ramirez S. (2007). Diuretic use, residual renal function, and mortality among hemodialysis patients in the Dialysis Outcomes and Practice Pattern Study (DOPPS). Am. J. Kidney Dis..

[107] Lee M.J., Park J.T., Park K.S., Kwon Y.E., Oh H.J., Yoo T.H., Kim Y.L., Kim Y.S., Yang C.W., Kim N.H. (2017). Prognostic Value of Residual Urine Volume, GFR by 24-hour Urine Collection, and eGFR in Patients Receiving Dialysis. Clin. J. Am. Soc. Nephrol..

[108] Hoek F.J., Korevaar J.C., Dekker F.W., Boeschoten E.W., Krediet R.T. (2007). Estimation of residual glomerular filtration rate in dialysis patients from the plasma cystatin C level. Nephrol. Dial. Transplant..

[109] Vilar E., Boltiador C., Wong J., Viljoen A., Machado A., Uthayakumar A., Farrington K. (2015). Plasma Levels of Middle Molecules to Estimate Residual Kidney Function in Haemodialysis without Urine Collection. PLoS ONE.

[110] Wong J., Sridharan S., Berdeprado J., Vilar E., Viljoen A., Wellsted D., Farrington K. (2016). Predicting residual kidney function in hemodialysis patients using serum beta-trace protein and beta2-microglobulin. Kidney Int..

[111] Shafi T., Michels W.M., Levey A.S., Inker L.A., Dekker F.W., Krediet R.T., Hoekstra T., Schwartz G.J., Eckfeldt J.H., Coresh J. (2016). Estimating residual kidney function in dialysis patients without urine collection. Kidney Int..

[112] Steubl D., Fan L., Michels W.M., Inker L.A., Tighiouart H., Dekker F.W., Krediet R.T., Simon A.L., Foster M.C., Karger A.B. (2019). Development and Validation of Residual Kidney Function Estimating Equations in Dialysis Patients. Kidney Med..

[113] van Olden R.W., van Acker B.A., Koomen G.C., Krediet R.T., Arisz L. (1995). Time course of inulin and creatinine clearance in the interval between two haemodialysis treatments. Nephrol. Dial. Transplant..

[114] Daugirdas J.T. (2021). Equations to Estimate the Normalized Creatinine Generation Rate (CGRn) in 3/Week Dialysis Patients With or Without Residual Kidney Function. J. Ren. Nutr..

[115] Colton C.K., Smith K.A., Merrill E.R., Friedman S. (1971). Diffusion of urea in flowing blood. AIChE J..

[116] Obi Y., Kalantar-Zadeh K., Streja E., Daugirdas J.T. (2018). Prediction equation for calculating residual kidney urea clearance using urine collections for different hemodialysis treatment frequencies and interdialytic intervals. Nephrol. Dial. Transplant..

[117] National Kidney Fundation (2001). NKF-K/DOQI Clinical Practice Guidelines for Peritoneal Dialysis Adequacy: Update 2000. Am. J. Kidney Dis..

[118] National Kidney Foundation (2015). KDOQI Clinical Practice Guideline for Hemodialysis Adequacy: 2015 Update. Am. J. Kidney Dis..

[119] Daugirdas J.T. (2016). Estimating Weekly Urine Flow Rate And Residual Kidney Urea Clearance: A Method To Deal With Interdialytic Variability. Semin. Dial..

[120] Kestenbaum B., Ix J.H., Gansevoort R., Granda M.L., Bakker S.J.L., Groothof D., Kieneker L.M., Hoofnagle A.N., Chen Y., Wang K. (2022). Population-Based Limits of Urine Creatinine Excretion. Kidney Int. Rep..

[121] Agarwal R., Delanaye P. (2019). Glomerular filtration rate: When to measure and in which patients?. Nephrol. Dial. Transplant..

[122] Delanaye P., Ebert N., Melsom T., Gaspari F., Mariat C., Cavalier E., Bjork J., Christensson A., Nyman U., Porrini E. (2016). Iohexol plasma clearance for measuring glomerular filtration rate in clinical practice and research: A review. Part 1: How to measure glomerular filtration rate with iohexol?. Clin. Kidney J..

[123] Shafi T., Levey A.S., Inker L.A., Schwartz G.J., Knight C., Abraham A.G., Eckfeldt J.H., Coresh J. (2015). Plasma Iohexol Clearance for Assessing Residual Kidney Function in Dialysis Patients. Am. J. Kidney Dis..

[124] Agarwal R., Bills J.E., Yigazu P.M., Abraham T., Gizaw A.B., Light R.P., Bekele D.M., Tegegne G.G. (2009). Assessment of iothalamate plasma clearance: Duration of study affects quality of GFR. Clin. J. Am. Soc. Nephrol..

[125] White C.A., Akbari A., Allen C., Day A.G., Norman P.A., Holland D., Adams M.A., Knoll G.A. (2021). Simultaneous glomerular filtration rate determination using inulin, iohexol, and (99m)Tc-DTPA demonstrates the need for customized measurement protocols. Kidney Int..

[126] Shafi T., Levey A.S. (2018). Measurement and Estimation of Residual Kidney Function in Patients on Dialysis. Adv. Chronic Kidney Dis..

[127] Sambataro M., Thomaseth K., Pacini G., Robaudo C., Carraro A., Bruseghin M., Brocco E., Abaterusso C., DeFerrari G., Fioretto P. (1996). Plasma clearance rate of 51Cr-EDTA provides a precise and convenient technique for measurement of glomerular filtration rate in diabetic humans. J. Am. Soc. Nephrol..

[128] Dowling T.C., Frye R.F., Fraley D.S., Matzke G.R. (1999). Comparison of iothalamate clearance methods for measuring GFR. Pharmacotherapy.

[129] Odlind B., Hallgren R., Sohtell M., Lindstrom B. (1985). Is 125I iothalamate an ideal marker for glomerular filtration?. Kidney Int..

[130] Seegmiller J.C., Eckfeldt J.H., Lieske J.C. (2018). Challenges in Measuring Glomerular Filtration Rate: A Clinical Laboratory Perspective. Adv. Chronic Kidney Dis..

[131] Yang Q., Li R., Zhong Z., Mao H., Fan J., Lin J., Yang X., Wang X., Li Z., Yu X. (2011). Is cystatin C a better marker than creatinine for evaluating residual renal function in patients on continuous ambulatory peritoneal dialysis?. Nephrol. Dial. Transplant..

[132] Mathew A.T., Fishbane S., Obi Y., Kalantar-Zadeh K. (2016). Preservation of residual kidney function in hemodialysis patients: Reviving an old concept. Kidney Int..

[133] D’Onofrio G., Simeoni M., Rizza P., Caroleo M., Capria M., Mazzitello G., Sacco T., Mazzuca E., Panzino M.T., Cerantonio A. (2017). Quality of life, clinical outcome, personality and coping in chronic hemodialysis patients. Ren. Fail..

[134] Okazaki M., Obi Y., Shafi T., Rhee C.M., Kovesdy C.P., Kalantar-Zadeh K. (2023). Residual Kidney Function and Cause-Specific Mortality Among Incident Hemodialysis Patients. Kidney Int. Rep..

[135] Hemodialysis Adequacy 2006 Work Group (2006). Clinical practice guidelines for hemodialysis adequacy, update 2006. Am. J. Kidney Dis..

[136] Rhee C.M., Obi Y., Mathew A.T., Kalantar-Zadeh K. (2018). Precision Medicine in the Transition to Dialysis and Personalized Renal Replacement Therapy. Semin. Nephrol..

[137] Li T., Wilcox C.S., Lipkowitz M.S., Gordon-Cappitelli J., Dragoi S. (2019). Rationale and Strategies for Preserving Residual Kidney Function in Dialysis Patients. Am. J. Nephrol..

[138] Patel S.M., Kang Y.M., Im K., Neuen B.L., Anker S.D., Bhatt D.L., Butler J., Cherney D.Z.I., Claggett B.L., Fletcher R.A. (2024). Sodium Glucose Co-transporter 2 Inhibitors and Major Adverse Cardiovascular Outcomes: A SMART-C Collaborative Meta-Analysis. Circulation.

[139] Heerspink H.J.L., Berger S., Gansevoort R.T., Renal Life Cycle Trial Investigators (2023). Will SGLT2 Inhibitors Be Effective and Safe in Patients with Severe CKD, Dialysis, or Kidney Transplantation. Clin. J. Am. Soc. Nephrol..

[140] Daugirdas J.T., Greene T., Rocco M.V., Kaysen G.A., Depner T.A., Levin N.W., Chertow G.M., Ornt D.B., Raimann J.G., Larive B. (2013). Effect of frequent hemodialysis on residual kidney function. Kidney Int..

[141] Rocco M.V., Daugirdas J.T., Greene T., Lockridge R.S., Chan C., Pierratos A., Lindsay R., Larive B., Chertow G.M., Beck G.J. (2015). Long-term Effects of Frequent Nocturnal Hemodialysis on Mortality: The Frequent Hemodialysis Network (FHN) Nocturnal Trial. Am. J. Kidney Dis..

[142] Liu S., Diao Z., Zhang D., Ding J., Cui W., Liu W. (2014). Preservation of residual renal function by not removing water in new hemodialysis patients: A randomized, controlled study. Int. Urol. Nephrol..

